# Accreditation and certification requirements for hernia centers and surgeons: the ACCESS project

**DOI:** 10.1007/s10029-018-1873-2

**Published:** 2019-01-23

**Authors:** F. Köckerling, A. J. Sheen, F. Berrevoet, G. Campanelli, D. Cuccurullo, R. Fortelny, H. Friis-Andersen, J. F. Gillion, J. Gorjanc, D. Kopelman, M. Lopez-Cano, S. Morales-Conde, J. Österberg, W. Reinpold, R. K. J. Simmermacher, M. Smietanski, D. Weyhe, M. P. Simons

**Affiliations:** 1 Department of Surgery, Center for Minimally Invasive Surgery, Academic Teaching Hospital of Charité Medical School, Vivantes Hospital, Neue Bergstrasse 6, 13585 Berlin, Germany; 2 Associate Clinical Head of Division (Surgery), Manchester University NHS Foundation Trust, Manchester, UK; 3 General and HPB Surgery and Liver Transplantations, Pancreas and Abdominal Wall Specialist, Universitair Ziekenhuis Gent, C. Heymanslaan 10, 9000 Ghent, Belgium; 4 General and Day Surgery Unit, Center of Research and High Specialization for the Pathologies of Abdominal Wall and Surgical Treatment and Repair of Abdominal Hernia, Milano Hernia Center, Instituto Clinico Sant’Ambrogio, University of Insurbria, Milan, Italy; 5 Chief Week Surgery Departmental Unit, Department of General, Laparoscopic and Robotic Surgery, A.O. Dei Colli Monaldi Hospital Naples, Naples, Italy; 6 Department of General, Visceral and Oncological Surgery, Wilhelminenspital, 1160 Vienna, Austria; 7 Surgical Department, Horsens Regional Hospital, Horsens, Denmark; 8 Unité de Chirurgie Viscérale, Hôpital Privé d’Antony, 1, Rue Velpeau, 92160 Antony, France; 9 Department of Surgery, Krankenhaus der Barmherzigen Brüder, Spitalgasse 26, 9300 St. Veit an der Glan, Austria; 10 Department of Surgery Emek Medical Center, Afula and the Rappaport Faculty of Medicine, Technion-Israel Institute of Technology, Haifa, Israel; 11 Abdominal Wall Surgery Unit, Department of General Surgery, Hospital Universitari Vall d’Hebron, Universitat Autònoma de Barcelona, Passeig Vall d’Hebron, 119-129, 08035 Barcelona, Spain; 12 Unit of Innovation in Minimally Invasive Surgery, University Hospital Virgen del Rocío, Av. Manuel Siurot, s/n, 41013 Seville, Spain; 13 Department of Surgery, Mora Hospital, 79285 Mora, Sweden; 14 Wilhelmsburger Krankenhaus Gross-Sand, Gross-Sand 3, 21107 Hamburg, Germany; 15 Department of Surgery, University Medical Center Utrecht, Heidelbergglaan 100, Utrecht, The Netherlands; 16 Department of General Surgery and Hernia Centre, Hospital in Puck, Medical University of Gdansk, Gdańsk, Poland; 17 School of Medicine and Health Sciences, University Hospital for Visceral Surgery, Pius-Hospital Oldenburg, Medical Campus University of Oldenburg, Georgstrasse 12, 26121 Oldenburg, Germany; 18 Department of Surgery, OLVG Hospital, Oosterpark 9, 1091 AC Amsterdam, The Netherlands

**Keywords:** Hernia center, Specialist hernia surgeon, Guidelines, Certification requirements, Accreditation

## Abstract

**Introduction:**

There is a need for hernia centers and specialist hernia surgeons because of the increasing complexity of hernia surgery procedures due to new techniques, more difficult cases and a tailored approach with an increasing public awareness demanding optimal treatment results. Therefore, the requirements for accredited/certified hernia centers and specialist hernia surgeons should be formulated by the international and national hernia societies, while taking account of the respective health care systems.

**Methods:**

The European Hernia Society (EHS) has appointed a working group composed of 18 hernia experts from all regions of Europe (ACCESS Group—Hernia Accreditation and Certification of Centers and Surgeons—Working Group) to formulate scientifically based requirements for hernia centers and specialist hernia surgeons while taking into consideration different health care systems. A consensus was reached on the key questions by means of a meeting, a telephone conference and the exchange of contributions. The requirements formulated below were deemed implementable by all participating hernia experts in their respective countries.

**Results:**

The ACCESS Group suggests for an adequately equipped hernia center the following requirements: (a) to be accredited/certified by a national or international hernia society, (b) to perform a higher case volume in all types of hernia surgery compared to an average general surgery department in their country, (c) to be staffed by experienced hernia surgeons who are beyond the learning curve for all types of hernia surgery recommended in the guidelines and are responsible for education and training of hernia surgery in their department, (d) to treat hernia patients according to the current guidelines and scientific recommendations, (e) to document each case prospectively in a registry or quality assurance database (f) to perform follow-up for comparison of their own results with benchmark data for continuous improvement of their treatment results and ensuring contribution to research in hernia treatment. To become a specialist hernia surgeon, the ACCESS Group suggests a general surgeon to master the learning curve of all open and laparo-endoscopic hernia procedures recommended in the guidelines, perform a high caseload and additionally to implement and fulfill the other requirements for a hernia center.

**Conclusion:**

Based on the above requirements formulated by the European Hernia Society for accredited/certified hernia centers and hernia specialist surgeons, the national and international hernia societies can now develop their own programs, while taking account of their specific health care systems.

## Introduction

“Medicine is continuing to evolve and this is true more so than ever before with a notable direct relation to several possible major factors including substantial changes in the health care systems, progressive scientific advances, an accelerated pace of discovery in biomedical science, heightened public awareness and demands and consequently expectations for transparency and accountability in health care” [1]. “Considering this fundamental, dynamic and rapid evolution, there could also be challenges to and changes in the well-established structure of self-regulating functions involving the medical profession” [1]. “Accrediting and certifying organizations, such as surgical societies, can and should play a major role in ensuring that society will continue to entrust self-governance to the medical profession by directly promoting and supporting consistent excellence in the performance of physicians and health care organizations” [1].

The medical profession being charged with the remit of self-regulation may well create a relative disbalance with its apparent, albeit responsible, governance presenting difficulties for any laypersons who wish to scrutinize any such self-governed professional body. Good examples of such self-regulation include the governance seen in medical societies, for example, in specialist breast and bariatric centers. The European Society of Breast Cancer Specialists has continued to produce updated and revised guidelines on the requirements of a specialist breast center which are based on the advances and evidence-based changes in contemporaneous clinical practice [2].

As a consequence, there is now evidence of improved patient outcomes in bariatric surgery centers that have since been accredited/certified according to the requirements of the German Society for General and Visceral Surgery [3]. In a systematic review of the literature, there is clear evidence which demonstrated that accreditation programs will improve clinical outcomes in a wide spectrum of clinical conditions [4].

Each year some 20 million inguinal hernia operations are performed worldwide with 350,000 and 100,000 ventral hernia operations in the US and Germany, respectively [5, 6].

Hernia surgery has become increasingly more complex over the past 25 years because of the introduction of novel endoscopic, but also open, techniques and of the plethora of medico-technical devices which are now available [5, 6]. Currently though, the lack of standardization for abdominal wall hernia repair has led to the existence of a multitude of techniques and even more options are available for prosthetic mesh selection but with little high-level evidence to suggest the type of technique and mesh to use [6].

Despite this, numerous evidence-based guidelines published by international hernia societies are endeavoring to keep abreast of these rapid developments [7–18].

However, analyses by hernia registries [19] demonstrate that the evidence-based guidelines compiled by the international hernia societies are not always implemented [20].

Although hard evidence that specialist hernia centers perform better than surgeons and/or surgical teams in general practice is scarce, it seems obvious in a subjective analysis in various settings that this mere fact holds some merit. Any subsequent data analysis of these respective centers and their results is compounded by the fact that no clear hernia centers are defined, but it is understood that arbitrary nomenclature is used such as “self-proclaimed” hernia center with so-called hernia specialists. There is therefore a need for accredited/certified hernia centers where hernia surgery is practiced according to the guidelines set by specialist certified hernia surgeons. In addition, these centers will be encouraged with specialization to be coupled with a measurable mastery of hernia surgical techniques as well as playing an active role in training and continuing education in the field of science in hernia surgery [5].

A credible accreditation/certification process for hernia centers will involve definitions of requirements and their verification by hernia societies that are interested in assuring the best possible quality of hernia surgery [5].

Examples of such accreditation are the introduction in 2014 of a three-stage accreditation/certification program for hernia centers in Germany conjointly by the German Hernia Society and the German Society of General and Visceral Surgery [5] and more recently in 2018 the Italian Society of Hernia and Abdominal Wall Surgery which defines the characteristics of accredited/certified hernia centers in Italy [21].

In September 2017, the European Hernia Society (EHS) commissioned an expert group from across Europe to compile evidence-based requirements for accredited/certified hernia centers. In the absence of any such evidence to date, the goal in this project was to seek an expert, transparent and coherent consensus.

## Methods

The EHS Board invited 18 hernia experts from across Europe to convene in the ACCESS Group (Access Group—Hernia Accreditation and Certification of Centers and Surgeons—Working Group). The group was entrusted with the task of formulating evidence-based scientific requirements for accredited/certified hernia centers and surgeons. Where evidence from systematic reviews and/or meta-analyses existed, the Prisma Grading was used [22]. The ACCESS group based inclusion into the final script only on material that reached a firm expert consensus without in some cases the necessary evidence, as it was understood there will be reliance on the very high expertise in the field of hernia that was made available. Any recommendations were formulated as “suggestion” for weak and “recommendation” for strong evidence.

Firstly; relevant key questions on this topic were formulated and collated through email exchange within the ACCESS Group. Then, the individual key questions were distributed for processing among the members of the Group. The findings of literature analyses were then presented at meetings held by the Group, debated and the answer to each key question compiled in the form of a consensual recommendation. A meeting was held on January 12, 2018 in Berlin followed by a telephone conference on June 26, 2018. The manuscript was circulated multiple times for corrections and proposals within the group.

At the end of the meeting and the telephone conference, the answers to the key questions were collated for publication.

## Results

### Arguments for and against hernia centers

#### Key questions 1, 2

Do we need hernia centers and specialist hernia surgeons?

What are the pros and cons of accredited/certified hernia centers?

What are the risks?


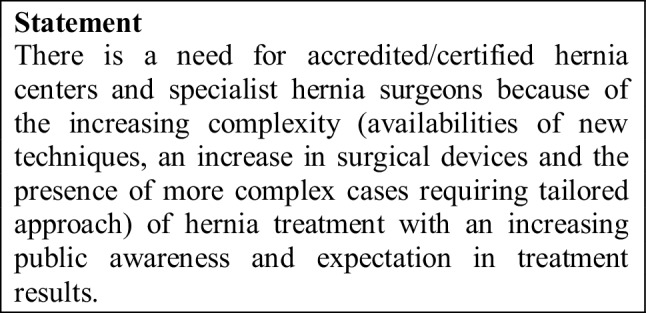


Most patients worldwide presenting with hernias are managed in general hospitals but because of a lack of definitions of specialist centers and/or surgeons, the exact percentages of those that are managed by “specialists” are almost impossible to obtain. For example, in the Netherlands, 645 out of 1500 surgeons performed inguinal hernia repair in 2016. The treatment was offered in all 87 hospitals with a range of 5–1200 repairs per year. 23/87 hospitals performed fewer than 200 repairs per year [23]. In Germany, there are more than 1200 hospitals with a department of general surgery, of which 82 are accredited/certified hernia centers with 2–3 accredited/certified hernia surgeons per center.

“General surgery has become increasingly fragmented into subspecialties and diseases previously treated by general surgeons are now managed by specialists” [24]. “The etiology of this evolution to sub-specialization in surgery is multifunctional, and this paradigm shift toward specialty surgery is almost certain to continue” [24]. “Numerous factors such as advances in surgical knowledge, techniques, and technology, as well as patient and physician preferences, have driven an increasing numbers of surgeons to specialization” [25].

“Hernia surgery has become increasingly more complex over the past 25 years because of the introduction of novel endoscopic and robotic, but also open techniques and of the plethora of medico technical devices” [26].

“The consideration of a herniorrhaphy among most general surgeons has changed. The past thinking “it’s just a hernia” is passé and has been replaced with the science-based consideration of patient-related factors, patient selection, anatomic application, fixation strength requirements, and healing considerations of biomaterials as well as truthful and true physical world-based postoperative activity restrictions” [26].

“This realm of greater understanding of abdominal wall problems and their repair has improved patient outcomes and delivered this form of surgery to a true specialty” [26].

“Differentiated use of the various techniques has been adapted as a “tailored approach” program and requires intensive engagement with, and extensive experience of, the entire field of hernia surgery. Eighty-two percent of experienced hernia surgeons are employing the “tailored approach” in hernia surgery” [27]. “The overall domain of hernia surgery has become more demanding” [5]. “A comparative study demonstrated that regardless of the surgical technique (open anterior mesh technique, plug technique, open posterior mesh technique, endoscopic technique), the recurrence rate is significantly higher for general surgeons who are not specialists in hernia surgery compared with hernia specialists (*p* < 0.0001)” [28]. “Therefore, there is a need for hernia centers and hernia specialists” [29].

The ACCESS Group is, of course, aware of the fact that in the future, too, the vast majority of hernia operations will be performed by non-specialist general surgeons. It is precisely because the number of hernia operations is so great that the findings of the ACCESS project should motivate as many general surgeons as possible to focus intensively on hernia surgery. That applies equally for primary care providers as well as for secondary/tertiary referral centers. Different levels of accredited/certified hernia centers (see “Key question 4” section) make it easier for all hospital levels to join the Hernia Center Program.

However, accreditation/certification as a hernia center cannot, on the one hand, guarantee optimum treatment for each hernia patient and, on the other hand, excellent hernia surgery can also be carried out without accreditation/certification as a hernia center. Therefore, as far as the quality of hernia surgery is concerned, accreditation/certification of hernia centers should not have medico-legal implications.

### Definition of a hernia center

#### Key question 3

What is the definition of an accredited/certified hernia center?

What could a definition be?


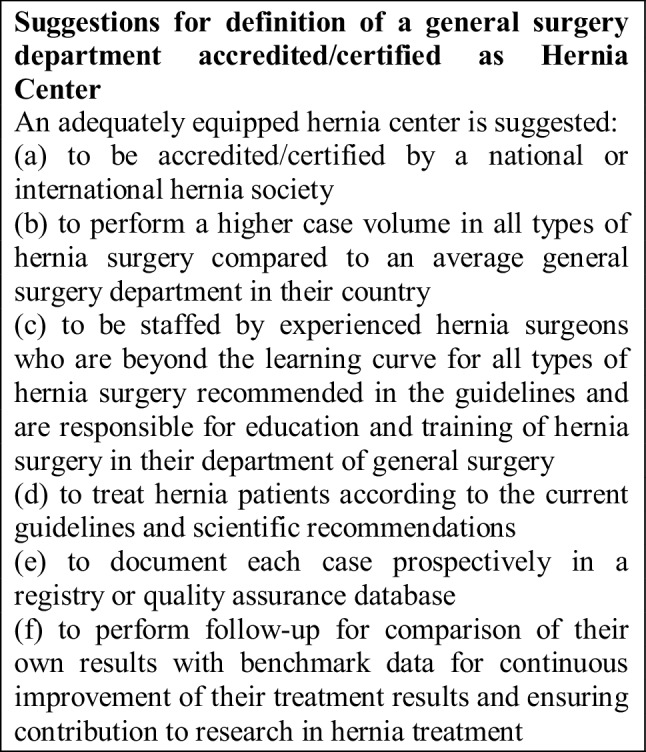


The establishment of a hernia center in accordance with the aforementioned requirements calls for, in addition to the choice and qualification of the responsible surgeons, appropriate support from the hospital administration to meet all the structural, equipment and personnel requirements.

#### Key question 4

Do we need different levels of accredited/certified hernia centers?


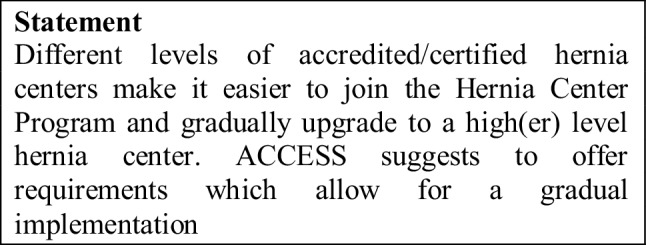


The Accreditation/Certification Program of the German Hernia Society (DHG) and the German Society of General and Visceral Surgery [5] provides for three levels:

- The German Hernia Society Seal of Participation in a hernia registry with presentation of one’s own results in comparison with existing benchmark data.
- The Competence Center which ensures compliance with structural and clinical requirements, case numbers as well as benchmark findings.
- The Reference Center which, in addition to meeting the requirements of the Competence Center, undertakes tasks in science, education and training for external surgeons. This system has the advantage that those hospitals and surgeons who are particularly active in hernia surgery can gradually implement and expand the requirements for a Reference Center. Requirements for the German Hernia Society Seal of Participation in a hernia registry are kept relatively low, so the use of the barest not so high requirements has enabled relatively high motivation of surgeons to participate in the hernia registry.

The Italian Society of Hernia and Abdominal Wall Surgery also propose three levels of accreditation/certification [21].

- First level certification is restricted to single surgeons.
- Second level certifications are referral centers for abdominal wall surgery
- Third level certifications are for highly specialized centers for abdominal wall surgery [21].

### Definition of a hernia specialist

#### Key questions 5, 6

Should the accreditation/certification process also be extended to specialist hernia surgeons particularly trained and experienced in hernia surgery?

What are the criteria that an accredited/certified specialist hernia surgeon needs to fulfill?


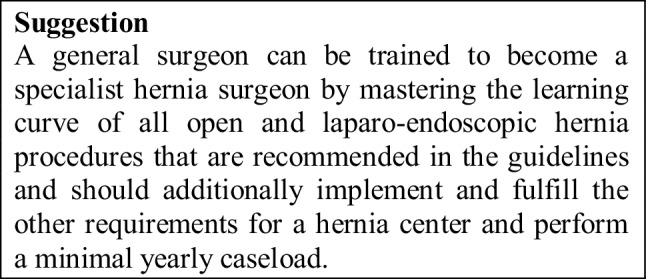


Accredited/certified hernia centers can offer an optimal environment for the education and training of hernia surgeons. If a surgeon works in a general surgery department and wants to become a specialist hernia surgeon, he/she should intend to perform at least 100 hernia operations per year, including assisting, and in addition assisting in several complex hernia repairs. General surgeons will be encouraged to undertake further training as hernia specialists in high-volume hernia institutions. In an accredited/certified hernia center, a surgeon should have played a ‘key role’ in at least 300 hernia surgical procedures within a period of 3 years and have met the other requirements to fulfill the prerequisites for a specialist hernia surgeon.

An example of caseload and volume can be found in the recommendations laid out for the attainment of a first level certification of a specialist hernia surgeon by the Italian Society of Hernia and Abdominal Wall Surgery: 120 inguinal hernia repairs (60 by open approach, 60 laparo-endoscopic, optional open preperitoneal) and 40 abdominal wall repairs (20 open, 20 laparoscopic) need to be performed with an annual caseload of 50 inguinal hernia repairs (25 open/25 laparo-endoscopic) and 50 incisional hernia repairs (25 open/25 laparoscopic) [21].

The numbers are representative of findings in the current literature pertaining mainly to the learning curve seen for the different procedures [21]. It is recommended and understood that to manage all eventualities in various clinical scenarios and perioperative complications, additional experience remains advisable.

Therefore, an accredited/certified specialist hernia surgeon should intend to have experience of at least 300 hernia operations, including 100 ventral and incisional hernia repairs.

### Institution for accreditation/certification of hernia centers

#### Key question 7

Should the national and international hernia societies be responsible for the accreditation/certification of hernia centers?


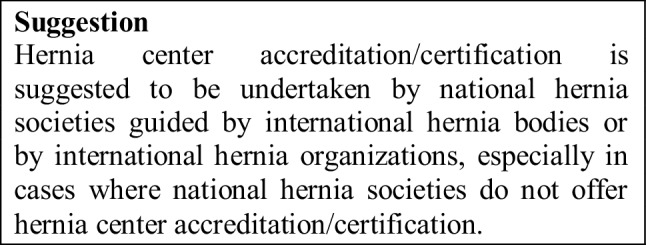


“The mission of the professional societies is primarily to provide education through information guided by evidence and considered best practice. Their relative influence flows from their continuing, highly visible function and moreover compulsory membership: this influence is necessary to steer publications in peer-reviewed journals, professional excellence, to raise public awareness and to make awards. Through their work, they help to define and set standards for their professional fields and to promote high standards of quality” [30]. “Medical societies play an important role in improving leadership in medicine” [31].

There are numerous examples that surgical societies implemented accreditation/certification programs for sub-specialties in surgery.

The American College of Surgeons (ACS) and the American Society for Metabolic and Bariatric Surgery (ASMBS) combined their respective national bariatric surgery accreditation programs into a single unified program to achieve one national accreditation standard for bariatric surgery centers: The Metabolic and Bariatric Surgery Accreditation and Quality Improvement Program [32].

The National Accreditation Program for Rectal Cancer is a newly developed initiative formed in collaboration with The OSTRICH Consortium (Optimizing the Surgical Treatment of Rectal Cancer) and the Commission on Cancer, a quality program of the American College of Surgeons [33]. “The Key to maintaining the highest standards of multidisciplinary care is accreditation of centers specializing in rectal cancer” [33].

The National Accreditation Program for Breast Centers represents a consortium of national, professional organization (e.g., American College of Surgeons, American Cancer Society, American Society of Clinical Oncology and others) dedicated to the improvement of the quality of care and monitoring of outcomes of patients with diseases of the breast [34].

In 2014, the German Hernia Society (DHG) and the German Society for General and Visceral Surgery introduced an accreditation/certification program for hernia centers [5]. A basic requirement for a credible certification process for hernia centers involves definition of the defined requirements and its subsequent verification by hernia societies [5]. In addition, in 2018, the Italian Society of Hernia and Abdominal Wall Surgery created a commission to define principles and structure of an accredited/certified hernia center [21].

Therefore, it is important that national and international hernia societies should be responsible for the accreditation/certification of hernia centers.

ACCESS (on behalf of the European Hernia Society) offers guidelines to develop and implement hernia centers and specialist hernia surgeons. National societies should be able to ensure that they take into consideration existing local factors.

### Auditing/re-auditing of a hernia center

#### Key question 8

What is the qualification of the auditors in the accreditation/certification process of hernia centers?


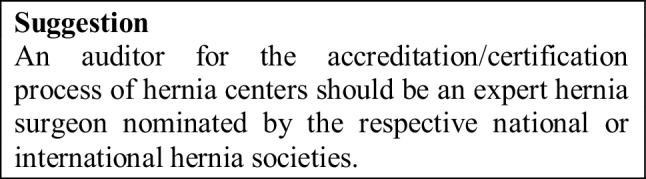


The importance of using a “reviewing/auditing” procedure that ratifies the requirements of the center and that it functions at the desired level depicting the adequate quality and standard is an unequivocal fact [35].

#### Key question 9

How long is the time interval after accreditation/certification of a hernia center for re-accreditation/re-certification?


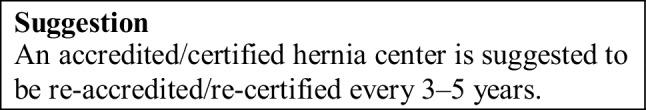


In the Quality Programs of the American College of Surgeons (Breast Center, Bariatric Surgery Center), the accredited/certified centers must completing a site visit every 3 years for re-accreditation/re-certification [36, 37].

#### Key question 10

What happens with the accreditation/certification of a hernia center, if a personnel or structural change occurs?


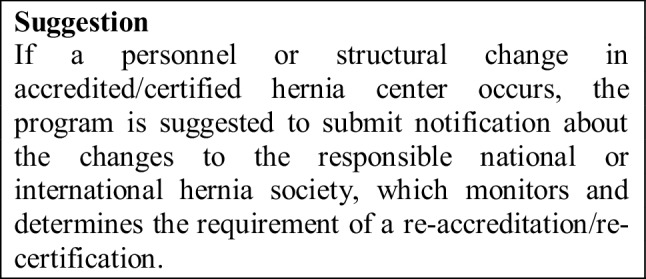


Quality program director changes must be notified to the American College of Surgeons 2018 [38]. The program must submit notification to the American College of Surgeons indicating the name, title, and contact information of the new program director along with the effective date of the appointment and whether the appointment is on an interim basis or permanent. The program must include documentation about the new program director’s qualifications and credentials which will be reviewed to ensure that the individual meets the requirements outlined in Standard 1.1 level of Responsibility and Accountability. The notice also must include the justification for the change [38].

### Equipping a hernia center

#### Key question 11

Which diagnostic tools need to be available in an accredited/certified hernia center?


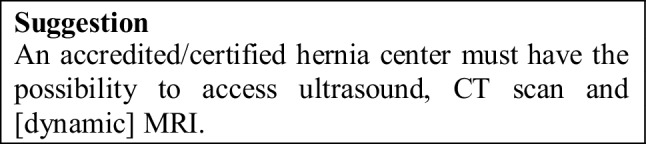


### Inguinal hernia

Using current best evidence in clinical decision making is always considered the gold standard. One way of presenting evidence to clinicians is through systematic reviews with summaries highlighting the highest impact on current evidence available. A key strategy to integrate the information of systematic reviews is through the development of clinical practice guidelines [7–18]. Recently, the International Guidelines for Groin Hernia Management have been published by the HerniaSurge Group [12] and in these guidelines four key questions (KQ) are put forward questioning the diagnostic modalities used for an inguinal hernia (Chap. 3).

The first KQ was which diagnostic modality is the most suitable for diagnosing groin hernias? The answer and recommendation is that clinical examination (CE) alone is adequate for providing evidence of a groin hernia. The level of evidence was low, however, a strong recommendation upgraded by the group was undertaken largely due to the fact that the history and clinical examination are all that are usually required to confirm the diagnosis of a clinically evident groin hernia.

The second KQ was which diagnostic modality is the most suitable for diagnosing patients with obscure pain or a ‘doubtful’ swelling? CE and ultrasound (US) used in combination is recommended for diagnosing patients with vague groin swelling or a possible occult groin hernia(s). Dynamic magnetic resonance image (MRI) or computed tomography (CT) can be considered for further evaluation if US is negative or non-diagnostic. The level of evidence was moderate and a strong recommendation upgraded by the group was undertaken.

The third KQ was which diagnostic modality is the most suitable for diagnosing recurrent groin hernias? CE and US combined is suggested as most suitable for confirming the diagnosis of a recurrent groin hernia. Dynamic MRI or CT can be considered for further evaluation if US is negative or non-diagnostic. The level of evidence was low and grade of recommendation was weak.

The fourth KQ was which diagnostic modality is the most suitable for investigating the cause of chronic pain after groin hernia surgery? Use of US-guided nerve blocks is suggested as helpful for investigating the cause of chronic pain after inguinal hernia surgery. US, CT or MRI scans are also helpful in identifying non-neuropathic causes of chronic groin pain (i.e., mesh-related pathologies, recurrent hernias and neuromas). The level of evidence was low and grade of recommendation was weak.

An accredited/certified hernia center must have available a variety of diagnostic modalities (i.e., expert clinical exam, US, CT and MRI) to cover the spectrum of investigations required to diagnose a groin hernia.

In the context of primary ventral and incisional hernias, preoperative imaging provides essential information for decision making [13, 17, 39]. One of the preoperative decisions that the surgeon needs to know is the feasibility of repair in terms of size of the defect and anterior abdominal wall, volume abdominal cavity/volume hernia, degree of central adiposity, grade of obesity and how effective weight loss would be, morphemic analysis (e.g., osseous margin, muscle contour), liquid collections, multiple defects, quality/quantity abdominal wall muscles and potential adherences. All these aspects should help the surgeon select which surgical approach will be required. Also, postoperative imaging provides essential information regarding any potential complications and possible recurrences [13, 17, 39]. In our learned opinion and in the context of primary ventral and incisional hernias, preoperative and postoperative (if needed) cross-sectional imaging (especially CT) play a critical role in assessing the likelihood of successful repair and by aiding in determining the optimal surgical approach.

In summary, an accredited/certified hernia center must therefore have available a variety of diagnostic modalities such as US, CT and MRI to cover the whole spectrum of preoperative and postoperative assessments of all the types of hernias that can present. It is also advisable that an abdominal wall surgeon work alongside a radiologist with special dedication to abdominal wall pathology.

#### Key question 12

Does an accredited/certified hernia center require an intensive care unit?


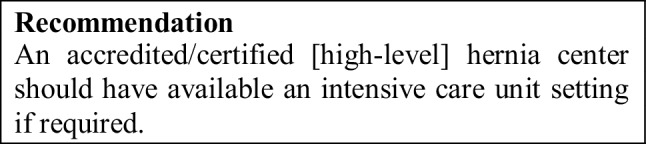


Below are listed the relevant citations in the literature which recommend the use of an intensive care unit in the care of patients undergoing hernia surgery.

In a prospective randomized trial comparing laparoscopic vs open incisional hernia repair, patients not amenable to extubation were admitted to the intensive care unit for observation and ventilator support [40].

In a study by Clarke [41] reporting about incisional hernia repair by fascial component separation, routine postoperative management entailed intensive care unit monitoring where appropriate.

Farooque et al. [42] presented a series with preoperative abdominal muscle elongation with botulinum toxin A for complex incisional ventral hernia repair. Postoperatively, patients required a period of ventilation in an intensive care unit.

The Accreditation/Certification Program of the Italian Society of Hernia and Abdominal Wall Surgery ask for availability of an intensive care unit in a high-level hernia center [21].

It is recognized that the management of large incisional hernia patients requires an intensive care unit.

#### Key question 13

Should an accredited/certified hernia center offer special consultation hours for hernia patients?


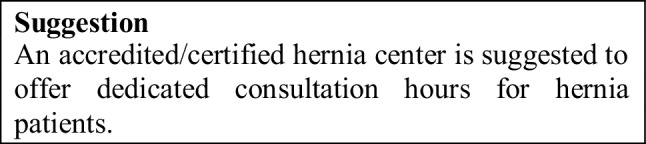


“Effective communication between primary care physicians and specialists regarding patient referrals and consultations is necessary for coordinated care, is important to patients and physicians and improves patient outcomes and physician satisfaction” [43] “Interspeciality communication is increasingly important because medical subspecialization and technological advances fragment care across numerous physicians” [43]. “The need for better dialogue and engagement between doctors and patients was also stressed though, together with the potential for a more collaborative relationship” [44]. To fulfill the demands of the primary care physicians and their hernia patients, regular pre- and postoperative visits in special consultation hours with the specialized surgeon are of great importance and value. An example of this system is noted in the requirements for the German and Italian certification programs for hernia centers which include weekly ‘dedicated’ consultation hours and outpatient clinics [5, 21].

#### Key question 14

Are regular morbidity conferences necessary for an accredited/certified hernia center?


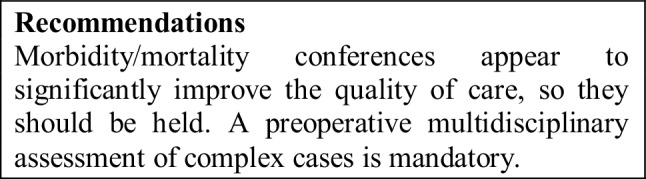


“Morbidity and mortality conferences have already been a representative example of both education and quality assurance within surgical departments” [45]. In a survey, there was a general agreement that surgical morbidity and mortality conferences are of good educational value as well as being effective in reducing potential errors [45]. “The majority of respondents expressed that evidence-based literature should be the primary basis of discussion, with comprehensive presentations that focus primarily on the analyses of the error” [45]. “The potential for learning from medical errors, complications and unanticipated outcomes is immense” [46]. “One basic premise is that greater recognition of prior mistakes offers the opportunity, in the future, to consequently avoid them if possible” [47].

Notwithstanding the value of learning from postoperative morbidity and mortality meetings, the preoperative assessment of a patient as well as prehabilitation has been shown to have a great influence on the inevitably preferred positive outcome for patients. “Pre-operative assessment should refer the patient, if necessary, for optimization of their health before surgery, e.g. to a primary care and/or a secondary care specialist” [48]. “Preoperative assessment of large ventral hernia defects is the cornerstone of success” [49]. “It allows for identification of factors that may preclude certain operative interventions and promotes presurgical steps to optimize a patient’s status before undergoing such a repair” [49].

#### Key question 15

Does a certified hernia center need to follow a special postoperative pain treatment regimen?


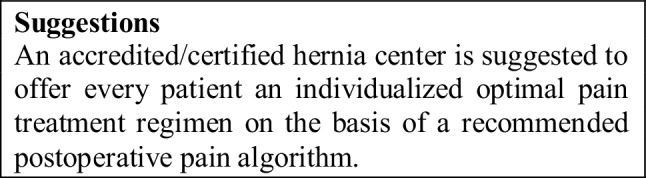


It is well-recognized that for an inguinal hernia repair, risk factors for chronic postoperative inguinal pain are present that include young age, female gender, high preoperative pain, early increased postoperative pain, recurrent and an open hernia repair [11]. Overall, the incidence of clinically significant chronic pain is in the 10–12% range, which decreases over time [11]. Debilitating chronic pain affecting normal daily activities or work ranges from 0.5 to 6% [11].

The intensity of perioperative pain has been suggested as a key risk factor [50]. Several other surgical, psychosocial and patient-related genetic and environmental risk factors have also been identified [50].

The use of multimodal analgesia (opioids, paracetamol, non-steroidal anti-inflammatory agents, gabapentinoids) is a recognized strategy and relied on the use of more than one class of analgesic agent and this has been advocated as a means to improve analgesia through either their additive or synergic effects, while reducing the opioid-related side effects [50].

Multimodal analgesia can be defined as a combination of an opioid and a non-opioid analgesia [50]. Whenever possible, multimodal pain management should be used [51]. Dosing regimens should be administered to optimize efficacy while minimizing the risk of adverse events [51]. The choice of medication, dose, route and duration of therapy should ideally be individualized [51].

### Quality assurance in a hernia center

#### Key question 16

Should an accredited/certified hernia center follow the guidelines of the international hernia societies?


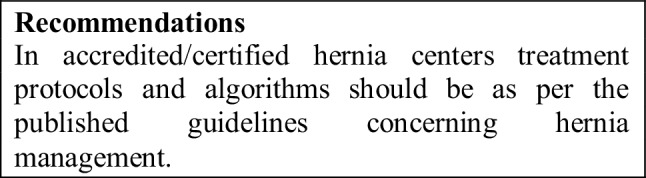


“Advances in medical, biomedical and health services research have reduced the level of uncertainty in clinical practice” [52]. “Clinical practice guidelines complement this progress by establishing standards of care backed by strong scientific evidence” [52]. “Clinical practice guidelines are statements that include recommendations intended to optimize care” [52]. “These statements are based on a systematic review of evidence and an assessment of the benefits and costs of alternative care options” [52]. “Clinical practice guidelines do examine the current state of clinical guidelines and consequently used to improve, enhance health care quality and inevitably patient outcomes” [52]. “Guidelines are designed as documents that help doctors understand the best way to diagnose, treat and even prevent diseases and conditions” [53]. “Clinical practice Guidelines can enhance both clinician and patient decisions –by translating complex scientific research findings into recommendations for clinical practice that are relevant to the individual patient encounter, instead of implementing a ‘one size fit all’ approach to patient care” [52]. “Clinical practice guidelines are now ubiquitous in our healthcare system” [52]. In the field of hernia surgery, numerous guidelines have been already published by respective responsible international hernia societies since 2009, which are also regularly validated and updated [7–18, 54–56].

In conclusion, to improve the quality of treatment in hernia patients and their outcomes, accredited/certified hernia centers should intentionally and conscientiously follow these peer-reviewed guidelines.

#### Key question 17

Is it a must for an accredited/certified hernia center to participate in a hernia quality assurance program or a defined registry?


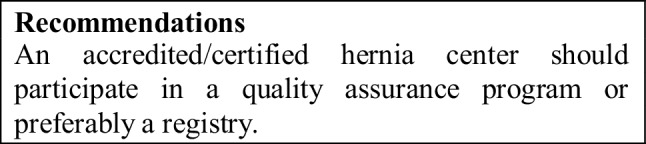


“Medical registries can serve different purposes but most are to the advantage of both patients and clinicians—for instance, as an important tool to monitor and improve quality of care or as a resource for research” [57].

“Establishing a nationwide groin hernia database leads to general improvement in outcomes and, due to the large number of patients on the dataset will allow analyses of specific sub-groups or complications which otherwise could not be obtained with the added objectivity as from single centers” [58]. “One cannot deny that the sharing and comparing data with similar colleagues and a measurement of one’s performance relative to the collective benchmark is likely to improve the safety and quality of health care rendered” [59]. “Registries can provide sound data needed by clinicians and organization to improve patient safety and quality of care” [60]. “Clinical registries do provide a clinically credible means of monitoring health care processes and outcomes” [61]. Registries are in particular suitable for evaluation of actual standard surgical practice examining both the level of an individual institution or on a national basis [62]. “While the seven hernia registries worldwide may differ in structure, together they contribute to raising the quality of hernia surgery” [19].

“Many hospitals unfortunately do not collect reliable data on their own adverse events, and you understandably cannot improve a hospital’s surgical quality if you are unable to measure it” [63].

For improvement in hernia patients and participation in research projects, it is recommended that accredited/certified hernia centers should participate in a quality assurance program or a defined registry. With widespread growing recognition that surgical outcomes vary by any one provider, surgeons and hospitals are increasingly being asked to provide evidence of the quality of care that they deliver [64].

On all levels of the accreditation/certification program of the German Hernia Society (DHG) and the German Society of General and Visceral Surgery (DGAV), participation in a hernia registry is a fundamental requirement with regular audit and presentations of the outcome measures [5]. The Italian program of accreditation/certification hernia centers does not appear to contain an obligatory participation in a hernia registry, but relies more so on trials arising from international centers [21].

### Clinical spectrum in a hernia center

#### Key question 18

In which techniques for inguinal hernia repair should an accredited/certified hernia center be proficient?


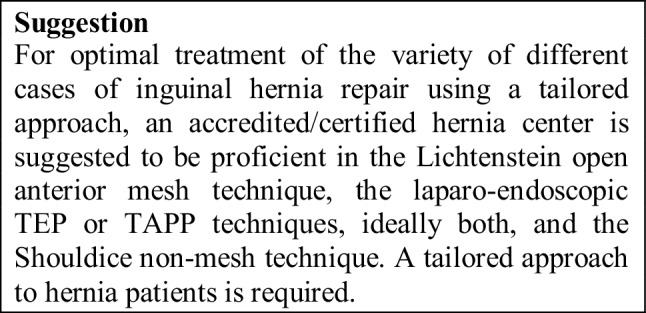


In the recently published new International Guidelines for groin hernia management of the HerniaSurge Group, a strong upgraded recommendation for a mesh-based repair of inguinal hernias is provided [12].

“Since a generally accepted technique which is suitable for all inguinal hernias does not exist, it is recommended that surgeons/surgical services provide an option of both anterior and posterior approaches” [12].

For an open anterior approach, the use of other implants (plug and patch, PHS, preperitoneal mesh) to replace the standard flat mesh in the Lichtenstein technique is currently not recommended [12]. In laparo-endoscopic posterior inguinal hernia repair, as TAPP and TEP have comparable outcomes, it is recommended that the choice of the technique should be based primarily on the surgeon’s skills, education and experience [12]. The Shouldice technique is recommended as the best non-mesh inguinal hernia repair, especially in cases where the patient refuses a mesh and/or after shared decision making [12].

#### Key question 19

In which ventral and incisional hernia repair technique should an accredited/certified hernia center be proficient?


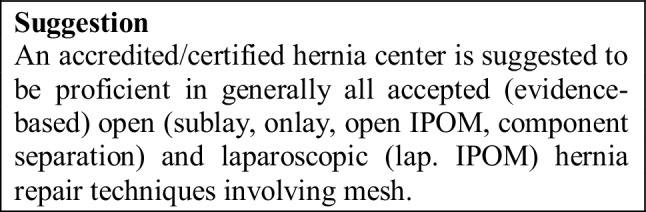


As laparoscopic ventral hernia repair has a lower rate of wound complications compared with open repair, numerous guidelines recommend that this technique is mainly reserved for defects not larger or less than 10 cm [13–15, 17, 18].

For open ventral and incisional hernia repairs, there are a number of options including sublay (retro-rectus, retromuscular), open IPOM (intraperitoneal or underlay), inlay (bridged or spanning the defect) [16]. An expert panel agreed on the basis of a systematic review that for open elective ventral and incisional hernia repair sublay mesh location is preferred [16]. However, open IPOM with intraperitoneal or underlay mesh positioning and also an onlay mesh positioning may be useful in certain settings [16]. Bridged open and inlay repairs should be avoided where possible, as these are associated with a higher rate of complications and recurrence [16]. Considering all the various types of component separation that are available with the endoscopic anterior and posterior approaches, using a mesh is recommended because of a lower recurrence rate and subsequent wound complications [16].

#### Key question 20

What is the spectrum of hernia types treated in an accredited/certified hernia center?


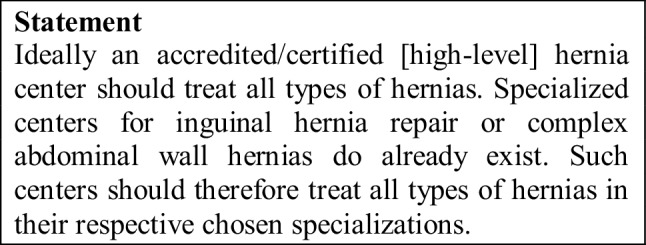


Rosen [65] focused on a team approach for providing the best care possible for patients with all types of hernias as his vision and strategic plan for the Digestive Disease Institute’s Hernia Center. “We see a wide spectrum of cases at our Hernia Center—from inguinal hernias that can be repaired laparoscopically on an outpatient basis, to incisional hernias with a 1–2 day length of stay, to ventral hernias that can be repaired with minimally invasive techniques if done right the first time” [65]. “We also specialize in complex open and repeat surgeries—including complex abdominal wall reconstruction—which are referred to us from all over the country and the world” [65].

The Hernia Institute of the University of Southern California delivers a high-quality interdisciplinary specialty care for patients with abdominal hernias, ranging from the simple to the most complex defects [66]. In addition mirroring this effect, the comprehensive Hernia Center of the University Hospitals Cleveland Medical Center, which is a team of highly skilled specialists provide today’s most advanced management of all types of hernias [67].

The comprehensive Hernia Center of Memorial Hospital of Rhode Island offers highly specialized care for people with all types of hernias, including all the latest treatments in hernia surgery, to ensure the best possible outcomes [68].

These above examples are both relevant and contemporaneous depictions of high-volume centers providing hernia care to all comers in terms of location, size and distribution of the abdominal wall defect.

### Caseloads in a hernia center

#### Key question 21

Should there be a minimum caseload per center per year?

If so, what should be the total caseload in an accredited/certified hernia center?


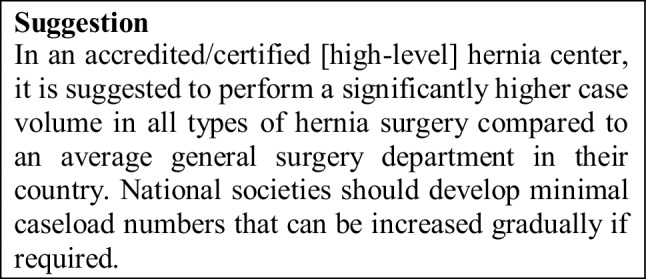


Surgeon volume is recognized as a net positive contributing factor for procedures that have a short length of stay, predominantly day case operations such as inguinal hernia surgery. But hospital factors, such as infrastructure and intensive care facilities become a more important prognostic denominator in procedures that are more complex and so require a more prolonged length of stay [69]. An observational study using data from 2009 to 2014 from inpatient treatment in German hospitals depicted this volume outcome trend for inguinal and femoral hernia repair with a greater volume resulting in reduced mortality (*n* = 897,000) with odds ratios of in-hospital death according to volume quintile of 1.00 for median annual volume of 68 cases, 0.94 (0.77–1.4) for median annual volume of 120 cases, 0.90 (0.72–1.11) for median annual volume of 160 cases, 0.83 (0.66–1.04) for median annual volume of 208 and 0.66 (0.51–0.86) for median annual volume of 312 [70]. Mortality in the very high-volume quintile was in the trend lower (0.07%, 95% CI 0.06–0.08) than in the very low-volume quintile (0.10%, 95 CI 0.09–0.12) [70].Based on complete national hospital discharge data for 25 types of inpatient treatments, the results confirmed the trends described above in volume–outcome relationships for many complex surgical procedures, as well as for some emergency conditions and low-risk procedures [70].

A review of the National Inpatient Sample database between 2008 and 2012 analyzed a total of 31,228 laparoscopic diaphragmatic hernia operations [71]. Pediatric, emergent, and open cases were excluded. The overall in-hospital mortality was 0.14% [71]. Using 10 cases per year as the volume threshold, low-volume hospitals had almost a twofold higher mortality compared to high-volume hospitals (0.23 vs 0.12%, respectively, *p* = 0.02). The authors concluded that there was a small but significant inverse relationship between the hospital’s case volume and mortality in laparoscopic diaphragmatic hernia repair [71].

The volume required in the German program for accreditation/certification of a high-level hernia center by the DHG and DGAV is at least 250 hernia operations per year, of which at least 50 must be incisional hernia operations, five complex hernias (e.g., parastomal hernia, component separation technique) and five hiatal hernias [5]. In the accreditation/certification program of the Italian Society of Hernia and Abdominal Wall Surgery for the third tier ‘High Specialization for Abdominal Wall Surgery’, the volume requirements for inguinal hernia repair are 150 procedures with 20 recurrent or scrotal hernias and 50 abdominal wall repairs with 20 complex cases [21].

#### Key question 22

Should there be a minimum caseload per surgeon?

What should be the caseload per surgeon in an accredited/certified hernia center?


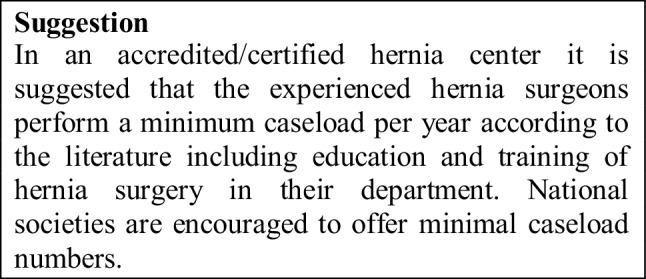


In a study from the Swedish Hernia Registry in 86,409 patients over a 15 year period, the re-operation rate for recurrence was significantly higher for surgeons who carried out 1–5 repairs a year than for surgeons who carried out more repairs [72].

In a retrospective review, a greater annual surgeon volume (> 30 vs 15–30 vs < 15) for inguinal hernia repair in totally extraperitoneal patch plasty technique was associated with improved outcomes as shown by the respective rates for intra- (1% vs 2.6% vs 5.6%) and postoperative (13% vs 27% vs 36%) complications, need for overnight stay (17% vs 23% vs 29%) and hernia recurrence (1% vs 4% vs 4.3%) (all p < 0.05) [73].

An analysis of the Herniamed Registry with 16,290 patients highlighted that low-volume surgeons (< 25 cases/year) had a significantly higher recurrence rate compared with high-volume surgeons (≥ 25 cases/year) following laparo-endoscopic inguinal hernia repair [74].

An analysis of the Statewide Planning and Research Cooperative System among 78,267 ventral hernia repairs and 124,416 inguinal hernia repairs demonstrated that the majority of variation in hernia recurrence was attributable to surgeon-level variation [75].This suggests that hernia recurrence may be an appropriate surgeon quality assurance metric [75].

In the first level certification of single surgeons in the certification program of the Italian Society of Hernia and Abdominal Wall Surgery, the volume requirements per year are defined as 50 inguinal hernia repairs (25 open, 25 laparo-endoscopic) and 50 incisional hernia repairs (25 open and 25 laparoscopic) [21].

### Results in a hernia center

#### Key question 23

What inguinal hernia repair results should be achieved in an accredited/certified hernia center?


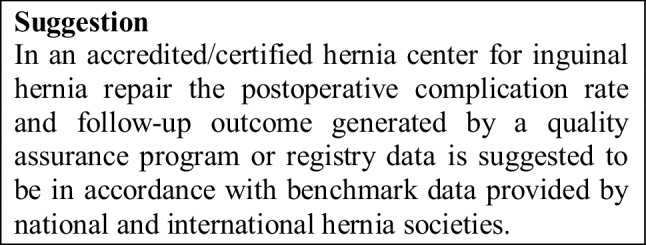


In a systematic review, the overall postoperative complication rate in 39 studies with 571,445 inguinal hernia repairs were evaluated [76]. Overall, 16,482 (2.9%) perioperative complications were reported [76]. The most commonly reported complications are bleeding (0.9%), wound infection (0.5%) and pulmonary and cardiovascular complications (0.2%), with 22% needing some form of re-intervention (Clavien-Dindo grade > IIIa) [76].

A registry-based, propensity score-matched comparison of 57,906 patients showed for the Lichtenstein technique a postoperative surgical complication rate of 3.4% with a complication-related re-operation rate of 1.1%, for the TEP technique of 1.7% and 0.8%, respectively, and for the TAPP technique 3.3% and 0.9%, respectively [77].

In the accreditation/certification program of hernia centers by the German Hernia Society (DHG) and the German Society of General and Visceral Surgery (DGAV), the recommended perioperative outcome 30 days postoperatively is < 5% rate for the total number of postoperative complications and < 2% rate for any complication-related reoperations [77].

The expected outcome according to the accreditation/certification program of the Italian Society of Hernia and Abdominal Wall Surgery is a morbidity < 10% and an infection rate of < 3% following inguinal hernia repair [21].

#### Key question 24

What outcome should be attainable for a primary ventral hernia repair in an accredited/certified hernia center?


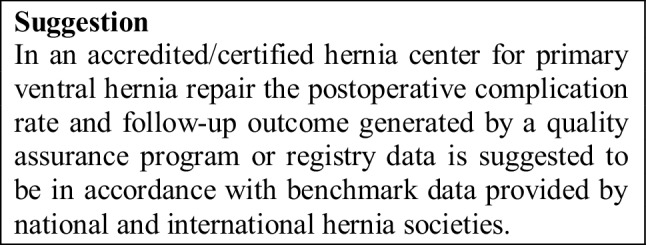


The 30-day postoperative complications after elective umbilical and/or epigastric hernia repair presented in non-nationwide and very heterogeneous studies are varied at 3–23% [78]. The national risk in Denmark of 30-day readmission following umbilical and epigastric hernia repair mainly due to the occurrence of a wound infection, hematoma, seroma, and pain is 5% with a complication-related re-operation rate of 0.3% [78]. In the Herniamed Registry, the 30-day postoperative surgical complication rates in 16,206 umbilical hernias was found to be 3.2% and in 3,757 epigastric hernias 3.5% [79]. The corresponding complication-related re-operation rates for umbilical and epigastric hernias were 1.0% and 1.2%, respectively [79].

#### Key question 25

What incisional hernia repair results should be achieved in an accredited/certified hernia center?


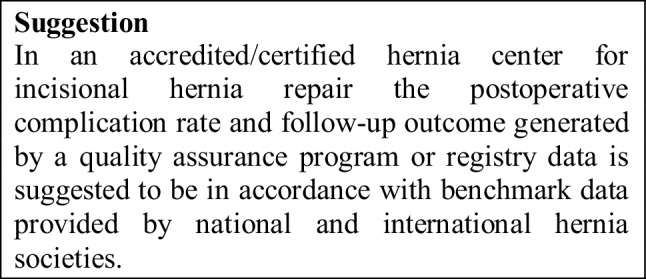


In a meta-analyses and systematic review of laparoscopic vs. open mesh repair for elective incisional hernia, the wound infection rates were 5.9% for laparoscopic and 8.5% for open [80].

A registry-based, propensity score-matched comparison of 9.907 patients with elective incisional hernia repairs the surgical postoperative complication rate within 30 days was 3.4% for laparoscopic IPOM and 10.5% for open sublay [81]. The complication-related re-operation rates were 1.5% for laparoscopic IPOM and 4.7% for sublay repair [81].

For the accreditation/certification program of hernia centers by the German Hernia Society (DHG) and the German Society for General and Visceral Surgery (DGAV), the required outcome rates for incisional hernia repair are less than 10% wound infection rate for open and less than 3% for laparoscopic repair with re-intervention rates of less than 10% for open and less than 3% for laparoscopic [5]. In the Italian certification program, the infection rate for abdominal wall hernia repair should also be lower than 10% and the recurrence rate at the first follow-up below 5% [21].

### Education and training in a hernia center

#### Key question 26

Does an accredited/certified hernia center need special training facilities with simulations to train hernia surgery?


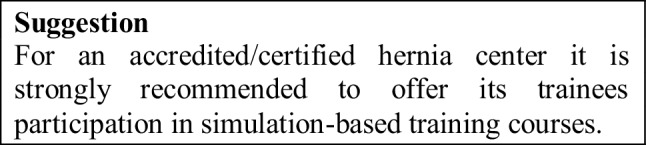


In a systematic review examining simulation-based training for laparoscopic surgery, 219 studies with 7138 trainees and 91 (42%) randomized controlled trials were included. For comparison with no intervention (*n* = 151 studies) pooled effect size favored simulation for outcomes of knowledge, skills time, skills process, skills product, behavior time, behavior process and patient effects (all *p* < 0.05) [82]. The authors concluded that simulation-based training for laparoscopic surgery of health professionals demonstrate large benefits when compared with no intervention and is also moderately more effective than non-simulation instructions [82].

In a randomized controlled trial, general surgery residents were randomized to mastery learning on standard practice after performing a baseline total extraperitoneal patch plasty (TEP) and reassessed during subsequent TEPs. Fifty residents performed 219 TEPs on 146 patients [83]. After training, TEPs performed by simulation-based mastery residents in training were undertaken with greater expediency than those in a standard practice residency program (34 ± 8 min vs 48 ± 14 min; *p* < 0.001) [83]. Intraoperative complications (peritoneal tear, procedure conversion), postoperative complications (urinary retention, seroma) and the need for overnight stay were less likely in the simulation-based mastery learning group adjusted odds ratio 0.14, [0.04–0] all *p* < 0.05 [83]. In conclusion, a simulation-based mastery learning curriculum decreased operative time, improved trainee performance and decreased intra- and postoperative complications as well as incidents needing overnight stay after laparoscopic TEP inguinal hernia repair [83].

According to the international guidelines for groin hernia management of the HerniaSurge Group, a goal-directed curriculum including review of the anatomy, procedure steps, intraoperative decision making and technical skills training shortens the learning curve for laparoscopic hernia repair and consequently will have a net benefit by improving patient outcomes [12].

The update of guidelines on laparoscopic (TAPP) and endoscopic (TEP) treatment of inguinal hernia by the International Endohernia Society recommends the availability of a simulation trainer to all surgical trainees to help improve their operative performance [10]. Currently, the trend is towards the use of box trainers over computer-assisted simulation for inguinal hernia repair training [10]. A proficiency-based curriculum for the available trainer tool should be established to improve patient outcomes [10].

There is also a positive correlation between laparoscopic ventral hernia repair simulation training and performance in the operating room [15].

#### Key question 27

Is access to the important hernia journals important for an accredited/certified hernia center?


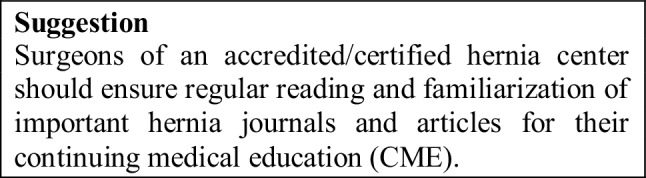


“Practicing medicine without reading is unthinkable and reading of contemporaneous journals and relevant research articles is extensively used in searching for information to solve clinical problems” [84]. “Journal reading is well established as an important source of continuing medical education of physicians, at least at the level of knowledge development” [85].

A meta-analysis suggests though that the effect of continuing medical education on a physician’s knowledge is of a medium one [86].

Subscription to the journal “Hernia” is therefore considered obligatory for all levels of an accredited/certified hernia center in the German program [5].

#### Key question 28

Should the surgeons of an accredited/certified hernia center regularly attend national and international meetings about hernia treatment?


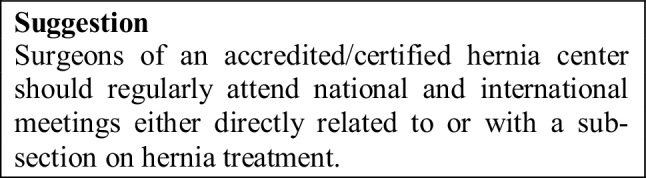


“The congresses enable health-care professionals to keep up-to-date with important research, learn directly from experiences and “trials and errors” of others, share best practices, and develop new skills and techniques. All of these have a direct impact on our daily clinical practice, helping us to improve safety and equality of care” [87]. “Exposure to other professionals may motivate the physicians to improve their performance and adapt continuous learning through the course of their careers” [87]. Demonstration of regular attendance at conferences is therefore to be considered an obligatory requirement for surgeons in an accredited/certified hernia center [5, 21].

#### Key question 29

Should an accredited/certified [high-level] hernia center actively participate in external education and training of surgeons in hernia surgery?


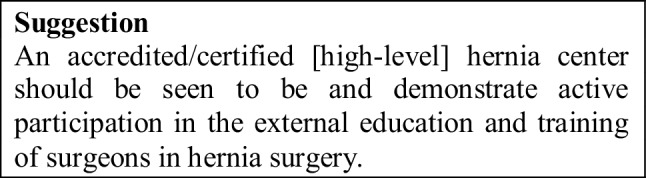


“Surgical education is by definition a lifelong process which starts with a solid training period and should be followed by high-quality continuing medical education (CME)” [88].

The potential providers of surgical education are surgical centers in their sub-specialty which have been identified as an accredited/certified center by their respective scientific societies [88]. Considered tertiary or high-level hernia centers that are accredited/certified by national or international hernia societies should be providers of external education and training of surgeons in hernia surgery. The accredited/certified high- or tertiary-level hernia centers must be actively involved in education and training of surgeons of external institutions [5, 21].

Accredited/certified (high level) hernia centers should help non-hernia specialists/centers to develop by cooperation, teaching, education and mentoring.

### Scientific activities of a hernia center

#### Key question 30

Should leading surgeons of accredited/certified hernia centers be members of the relevant scientific national and international hernia societies?


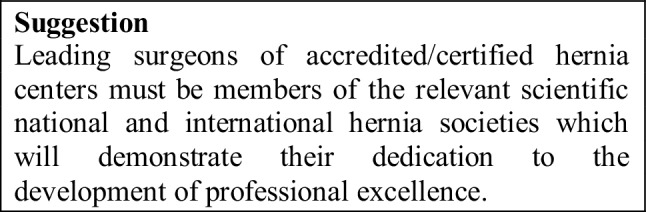


“Many scientific societies were founded to support the single disciplines for which they are named” [89].

“The mission of the scientific societies is primarily providing information through education, to publish in scientific journals, to develop professional excellence, to raise public awareness and to make awards” [89]. “Through this work, they help to define and set standards for their professional fields and to promote high standards of quality through awards and other forms of recognition”, for example, guidelines [89].

“Professional medical societies serve several functions that may benefit society, the medical profession, and individual members” [90].

“Participation can vary from membership to leadership” [90]. “Members may want to seek advice from senior colleagues to guide them in their research or for their own academic advancements” [31].

Accredited/certified hernia centers of all levels in the German program must be full members of the German Hernia Society (DHG) and the European Hernia Society (EHS) [5]. In the Italian program, all certified hernia surgeons and the leading surgeons of an accredited/certified hernia center must be members of the Italian Society of Hernia and Abdominal Wall Surgery, which is the Italian chapter of the European Hernia Society [21].

The leading surgeons of an accredited/certified hernia center should therefore be at least a member of the international hernia society if there is no national equivalent organization.

#### Key question 31

Should an accredited/certified [high-level] or tertiary hernia center participate actively in scientific projects of hernia treatment?


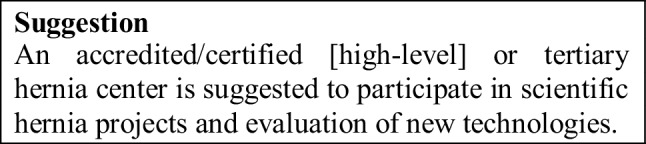


“Cooperation among a diverse group of stakeholders—including research sponsors (industry, academia, government, nonprofit organizations, and patient advocates), clinical investigators, patients, payers, physicians, and regulators—is necessary in conducting a clinical trial today” [91]. “The fewer physicians are involved in developing and implementing clinical trials, the less scientific the practice of medicine will be” [91]. “There is data to reflect that there is a disappointing trend that fewer professionals are undertaking research than in the past” [91]. Therefore, hernia centers accredited/certified by national or international hernia societies as high-level or tertiary institution should ideally be encouraged to actively participate in the scientific evaluation of their specific field with quality audit and research. In the German and Italian accreditation/certification programs, high level hernia centers are obliged to participate in hernia studies and research projects [5, 21].

#### Key question 32

Should surgeons of an accredited/certified [high-level] or tertiary hernia center regularly attend national and international meetings about hernia treatment, and present oral, video or poster presentations?


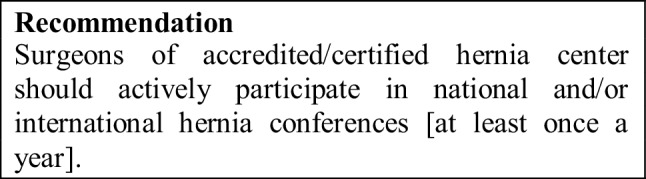


“The need for continuing self-improvement is the one element that is consistently seen as being central to professionalism across all disciplines” [85].

A meta-analysis of continuing medical education effectiveness demonstrated a better effect of active vs. passive participation in conferences [86]. Continuous medical education, especially in the active form, is likely to have an effect on a physician’s knowledge, performance and may also help improve patient outcomes [86]. Therefore, surgeons of accredited/certified hernia center should give—at least once a year—oral presentations or present videos/posters at national and/or international hernia conferences. In the Italian certification program considered high-level or tertiary hernia centers must participate at the annual congress of the European Hernia Society with abstracts and presentations [21]. For the German certification program, at least two papers or posters at national and international hernia conferences must be presented by high-level or tertiary hernia centers per year [5].

## Conclusions

Within the framework of the ACCESS project, a group of 18 hernia experts nominated by the European Hernia Society have formulated consensual recommendations and suggestions of requirements for accredited/certified hernia centers and specialist hernia surgeons. Even if the scientific evidence for these requirements is relatively low, experiences from other areas of medicine can be extrapolated to hernia surgery. This means that sufficient valid recommendations and suggestions are available for all key questions. Because of the differences in the health care systems of the various countries from which the participating hernia experts come, it is not possibly to stipulate specific numbers in the requirements. The view among working group members was that framework recommendations and suggestions should be agreed for the requirements, whereby the requirements would then be later supplemented with actual figures by the national and international hernia societies for the individual countries or continents. Hence, the recommendations and suggestions presented here are minimal requirements which can later be expressed in more concrete terms and expanded by the national and international hernia societies. However, already observance of the minimal requirements set out here for accredited/certified hernia centers and specialist hernia surgeons is likely to improve the quality of hernia surgery due to the experiences gained in other areas of surgical medicine. In view of the increasing complexity of hernia surgery, this development should be driven forward as far as possible by all national and international hernia societies. Therefore, based on the minimum requirements formulated here, all national and international hernia societies are called upon to develop and implement programs in their own countries for the accreditation/certification of hernia centers and specialist hernia surgeons. This also helps to meet the public expectation invested by the national and international hernia societies for optimization of patient treatment.

## References

[1] Bauchner H, Fontanarose PB, Thompson AE (2015). Professionalism, governance, and self-regulation of medicine. JAMA.

[2] Wilson ARM, Marotti L, Bianchi S (2013). The requirements of a specialist breast centre. Eur J Cancer.

[3] Stroh C, Köckerling F, Lange V (2017). Does certification as bariatric surgery center and volume influence the outcome on RYGB-data analysis of German bariatric surgery registry. Obes Surg.

[4] Alkhenizan A, Shaw C (2011). Impact of accreditation on the quality of health care services: a systematic review of the literature. Ann Saudi Med.

[5] Köckerling F, Berger D, Jost JO (2014). What is a certified hernia center? The example of the German Hernia society of general and visceral surgery. Front Surg.

[6] Krpata DM, Haskins IN, Rosenblatt S (2018). Development of a disease-based hernia program and the impact on cost for a hospital system. Ann Surg.

[7] Simons MP, Aufenacker T, Bay-Nielson M (2009). European hernia society guidelines on the treatment of inguinal hernia in adult patients. Hernia.

[8] Miserez M, Peeters E, Aufenacker T (2014). Update with level 1 studies of the European Hernia Society guidelines on the treatment of inguinal hernia in adult patients. Hernia.

[9] Bittner R, Arregui ME, Bisgaard T (2011). Guidelines for laparoscopic (TAPP) and endoscopic (TEP) treatment of inguinal Hernia [International Endohernia Society (IEHS)]. Surg Endosc.

[10] Bittner R, Montgomery MA, Arregui E (2015). Update of guidelines for laparoscopic (TAPP) and endoscopic (TEP) treatment of inguinal hernia [International Endohernia Society (IEHS)]. Surg Endosc.

[11] Poelman MM, van den Heuvel B, Deelder JD (2013). EAES consensus development conference on endoscopic repair of groin hernias. Surg Endosc.

[12] The HerniaSurge Group (2018). International guidelines for groin hernia management. Hernia.

[13] Bittner R, Bingener-Casey J, Dietz U (2014). Guidelines for laparoscopic treatment of ventral and incisional abdominal wall hernias (International Endohernia Society (IEHS))-part 1. Surg Endosc.

[14] Bittner R, Bingener-Casey J, Dietz U (2014). Guidelines for laparoscopic treatment of ventral and incisional abdominal wall hernias (International Endohernia Society (IEHS))-part 2. Surg Endosc.

[15] Bittner R, Bingener-Casey J, Dietz U (2014). Guidelines for laparoscopic treatment of ventral and incisional abdominal wall hernias ((International Endohernia Society (IEHS))-Part III. Surg Endosc.

[16] Liang MK, Holihan JL, Itani K (2017). Ventral hernia management: expert consensus guided by systematic review.. Ann Surg.

[17] Earle D, Roth JS, Saber A (2016). SAGES guidelines for laparoscopic ventral hernia repair. Surg Endosc.

[18] Silecchia G, Campanile DC, Sanchez L (2015). Laparoscopic ventral/incisional hernia repair: updated consensus development conference based guidelines [corrected]. Surg Endosc.

[19] Kyle-Leinhase I, Köckerling F, Jørgensen LN (2018). Comparison of hernia registries: the CORE project. Hernia.

[20] Köckerling F, Bittner R, Kuthe A et al (2017) Laparo-endoscopic versus open recurrent inguinal hernia repair: should we follow the guidelines? Surg Endosc(8):3168–3185. 10.1007/s00464-016-42-7 10.1007/s00464-016-5342-7 PMC5501902 27933397

[21] Stabilini C, Cavallaro G, Bocchi P (2018). Defining the characteristics of certified hernia centers in Italy: the Italian Society of Hernia and Abdominal Wall Surgery workgroup consensus on systematic reviews of the best available evidences. Int J Surg.

[22] Moher D, Liberati A, Tetzlaff J (2009). Preferred reporting items for systematic reviews and meta-analyses: the PRISMA statement. PLoS Med.

[23] Simons MP (2018) Personal communication

[24] Bruns SD, Davis BR, Aram ND (2014). The subspecialization of surgery: a paradigm shift. J Gasrointest Surg.

[25] Kulaylat AN, Zheng F, Bittner KUYS J. G (2013) Early surgical subspecialization: a new paradigm? Part II. American College of Surgeons. http://bulletin.facs.org/2013/08/early-surgical-subspecializaton-a-new-paradigm/. Accessed 10 May 2018 24205575

[26] Roll S (2012). A global vision for hernia repair improvement. Gen Surg News.

[27] Morales-Conde S, Socas M, Fingerhut A (2009). Endoscopic surgeons’ preferences for inguinal hernia repair: TEP, TAPP or OPEN. Surg Endosc.

[28] Gilbert AI, Graham MF, Zoung J (2006). Closer to an ideal solution for inguinal hernia repair: comparison between general surgeons and hernia specialists. Hernia.

[29] Miller G (2010). Hernia centers of excellence?. Gen Surg News.

[30] National Academy of Science (2018) Chapter: 7 the role of professional societies. NAP edu.10766. https://www.nap.edu/read/11153/chapter/9. Accessed 15 Apr 2018

[31] Raoof S (2013) Medical societies’ role in improving leadership in medicine. https://www.kevinmd.com/blog/2013/09/medical-cocieties-role-improving-leadership-in-medicine. Accessed 15 Apr 2018

[32] The American College of Surgeons (ACS) (2016) Metabolic and bariatric surgery accreditation and quality improvement program. http://www.facs.org/quality-programs/mbsagip. Accessed 15 Apr 2018

[33] Katerina W (2017). Rising the bar of rectal cancer: the national accreditation program for rectal cancer. Colorec Cancer.

[34] The American College of Surgeons (2018) National accreditation program for breast centers. http://www.facs.org/quality-programs/napbc/about. Accessed 12 July 2018

[35] Güler SA, Güllüoglu BM (2014). Quality assurance in breast health care and requirement for accreditation in specialized units. J Br Health.

[36] American College of Surgeons (2018) NAPBC accreditation. https://www.facs.org/quality-programs/napbc/accreditaton. Accessed 10 May 2018

[37] American College of Surgeons (2018) Initial applicants for accreditation. https://www.facs.org/quality-programs/mbsaqip/apply. Accessed 10 May 2018

[38] NAPBCAmerican College of Surgeons (2018) Breast program director change notification. http://www.napbc-breast.org. Accessed 10 May 2018

[39] Parikh KR, Al-Hawary M, Millet JD, Burney R, Finks J, Maturen K (2017). Incisional hernia repair: what the radiologist needs to know. AJR Am J Roentgenol.

[40] Eker H, Hanson B, Buunen M (2013). Laparoscopic vs open incisional hernia repair. A randomized clinical trial. JAMA Surg.

[41] Clarke J (2009). Incisional hernia repair by fascial component separation: results in 128 cases and evolution of technique. Am J Surg.

[42] Farooque F, Jacombs A, Roussos E (2015). Preoperative abdominal muscle elongation with botulinum toxin A for complex incisional ventral hernia repair. ANZ J Surg.

[43] O’Malley AS, Reschovsky JD (2011). Health Care Reform. Referral and consultation communication between primary care and specialist physicians. Arch Intern Med.

[44] British Medical Association (2017) The changing face or medicine and the role of doctors in the future. Presidential project http://www.bma.org.uk. Accessed 12 July 2018

[45] Gore DC (2006). National survey of surgical morbidity and mortality conferences. Am J Surg.

[46] Kravet SJ (2006). Morbidity and mortality conference, grand rounds, and the ACGME’s core competencies. J Gen Intern Med.

[47] Epstein NE (2012). Morbidity and mortality conferences: their educational role and why we should be there. Surg Neurol Int.

[48] NHS Modernisation Agency (2018) National good practice guidance on pre-operative assessment for inpatient surgery. http://www.hello.nhs.uk/documents/Preoperative%20assessment%20guidance%20for%20inpatient.pdf. Accessed 12 July 2018

[49] Trujillo CN, Fowler A, Al-Temimi MH (2018). Complex ventral hernia: a review of past to present. Perm J.

[50] Wu C, Raja S (2011). Treatment of acute postoperative pain. Lancet.

[51] American Society of Anesthesiologists Task Force on Acute Pain Management (2012). Practice guidelines for acute pain management in the perioperative setting: an updated report by the American Society of Anesthesiologists task force on acute pain management. Anesthesiology.

[52] Shekelle P et al (2011) Clinical practice guidelines we can trust. https://www.ncbi.nlm.nih.gov/books/NBK209539. https://uptodate.com/contents/overview-of-clinical-practice-guidelines. https://www.ncbi.nlm.nih.gov/books/NBK209538. Accessed 3 May 2018

[53] American Heart Association News. What is a medical guideline, and how is it created? https://news.heart.org/what-is-a-medical-guideline-and-how-is-ti-created/. Accessed 3 May 2018

[54] Antoniou AA, Agresta F, Garcia Alamino JM (2018). European Hernia Society guidelines on prevention and treatment of parastomal hernias. Hernia.

[55] Muysoms FE, Antoniou SA, Bury K (2015). European Hernia Society guidelines on the closure of abdominal wall incisions. Hernia.

[56] Kohn GP, Price RR, De;ester SR (2013). Guidelines for the management of hiatal hernia. Surg Endosc.

[57] Arts D, de Keizer N, Scheffer G-J (2002). Defining and improving data quality in medical registries: a literature review, case study, and generic framework. J Am Med Inf Assoc.

[58] Kehlet H, Bay-Nielsen M (2008). Nationwide quality improvement of groin hernia repair from the Danish Hernia Database of 87,840 patients from 1998 to 2005. Hernia.

[59] Lee MJ (2013). Safety in surgery: the role for registries. Clin Orthop Relat Res.

[60] McNeil JJ, Evans SM, Johnson NP, Cameron PA (2010). Clinical-quality registries: their role in quality improvement. MJA.

[61] Evans SM, Scott IA, Johnson NP, Cameron PA, McNeil JJ (2011). Development of clinical-quality registries in Australia: the way forward. Med J Aust.

[62] Glicklich RE, Dreyer NA, Leavy MB (2014) Registry design. In: Glicklich RE et al Registries for evaluating patient outcomes: a user’s guide. https://www.ncbi.nlm.nih.gov/books/NBK208632/. Accessed 3 May 2018

[63] Landro L (2015). How to make surgery safer. Wall Street J.

[64] Birkmeyer JD, Dimick JB, Birkmeyer NJO (2004). Measrung the quality of surgical care: structure, process, or outcomes?. J Am Coll Surg.

[65] Q&A with Dr. Michael Rosen: New Hernia Center Director. https://consultqd.clevelandclinic.org/qa-with-dr-michael-rosen-new-hernia-center-director. Accessed 8 May 2018

[66] USC University of Southern California. The Hernia Institute of USC. http://www.surgery.usc.edu/(uppergi-general/herniainstitute.html. Accessed 8 May 2018

[67] University Hospitals Cleveland Medical Center. Comprehensive Hernia Center. http://www.uhhospitals.org/cleveland/services/surgery/our-divisions/general-and-gastrointestinal-surgery. Accessed 8 May 2018

[68] Memorial Hospital of Rhode Island (2014) Comprehensive Hernia Center. Rhode Island Med J 63 24960919

[69] Morche J, Mathes T, Pieper D (2016). Relationship between surgeon volume and outcomes: a systematic review of systematic reviews. Syst Rev.

[70] Nimptsch U, Mansky T (2017). Hospital volume and mortality for 25 types of inpatient treatment in German hospitals: observational study using complete nation data from 2009 to 2014. BMJ Open.

[71] Whealon MD, Blondet JJ, Gahagan JV (2017). Volume and outcomes relationship in laparoscopic diaphragmatic hernia repair. Surg Endosc.

[72] Nordin P, van der Linden W (2008). Volume of procedures and risk of recurrence after repair of groin hernia: national register study. BMJ.

[73] AlJamal YN, Zendejas B, Gas Becca L (2016). Annual surgeon volume and patient outcomes following laparoscopic totally extraperitoneal inguinal hernia repairs. J Laparoendosc Adv Surg Tech.

[74] Köckerling F, Bittner R, Kraft B (2017). Does surgeon volume matter in the outcome of endoscopic inguinal hernia repair?. Surg Endosc.

[75] Aquina CT, Fleming FJ, Becerra AZ (2017). Explaining variation in ventral and inguinal hernia repair outcomes: a population-based analysis. Surgery.

[76] Weyhe D, Tabriz N, Sahlmann B, Uslar VN (2017). Risk factors for perioperative complications in inguinal hernia repair—a systematic review. Innov Surg Sci.

[77] Köckerling F, Bittner R, Kofler M (2017). Lichtenstein versus total extraperitoneal patch plasty versus transabdominal patch plasty technique for primary unilateral inguinal hernia repair. Ann Surg.

[78] Helgstrand F (2016). National results after ventral hernia repair. Dan Med J.

[79] Köckerling F, Schug-Paß C, Adolf D (2015). Is pooled data analysis of ventral and incisional hernia repair acceptable?. Front Surg.

[80] Awaiz A, Rahman F, Hossain MB (2015). Meta-analysis and systematic review of laparascopic versus open mesh repair for elective incisional hernia. Hernia.

[81] Köckerling F, Simon T, Adolf D (2019). Laparoscopic IPOM versus open sublay technique for elective incisional hernia repair: a registry-based, propensity score-matched comparison of 9907 patients. Surg Endosc.

[82] Zendejas B, Brydges R, Hamstra S, Cook D (2013). State of the evidence on simulation-based training for laparoscopic surgery: a systematic review. Ann Surg.

[83] Zendejas B, Cook D, Bingener J (2011). Simulation-based mastery learning improves patient outcomes in laparoscopic inguinal hernia repair: a randomized controlled trial. Ann Surg.

[84] Holm H (2000). Should doctors get CME points for reading? Yes: relaxing documentation doesn’t imply relaxing accountability. BMJ.

[85] Davidoff F (1997). Continuing medical education resources. J Gen Intern Med.

[86] Mansouri M, Lockyer J (2007). A meta-analysis of continuing medical education effectiveness. J Contin Educ Health Prof.

[87] Mishra S (2016). Do medical conferences have a role to play? Sharpen the saw. Indian Heart J.

[88] Peracchia A (2001). Surgical education in the third millennium. Ann Surg.

[89] The National Academic Press OPENBOOK, Facilitating Interdisciplinary Research (2005) Chapter: 7 The Role of Professional Societies. NAP.edu/1076. https://www.nap.edu/read/11153/chapter/9Accessed 15 Apr 2018

[90] Beck D (2011). Role of professional societies in career development. Clin Colon Rectal Surg.

[91] National Academy of Sciences (2010) Challenges in clinical research. NCBI Bookshelf. A service of the National Library of Medicine Institutes of Health. Bookshelf ID: NBK50888

